# The interplay of helminthic neuropeptides and proteases in parasite survival and host immunomodulation

**DOI:** 10.1042/BST20210405

**Published:** 2022-01-25

**Authors:** Rimanpreet Kaur, Naina Arora, Meera G. Nair, Amit Prasad

**Affiliations:** 1 School of Basic Sciences, Indian Institute of Technology Mandi, Mandi, Himachal Pradesh 175005, India; 2 Division of Biomedical Sciences, University of California Riverside, Riverside, CA 92521, U.S.A.

**Keywords:** helminth, immunomodulation, neuropeptide

## Abstract

Neuropeptides comprise a diverse and broad group of neurotransmitters in vertebrates and invertebrates, with critical roles in neuronal signal transduction. While their role in controlling learning and memory in the brains of mammals is known, their extra-synaptic function in infection and inflammation with effects on distinct tissues and immune cells is increasingly recognized. Helminth infections especially of the central nervous system (CNS), such as neurocysticercosis, induce neuropeptide production by both host and helminth, but their role in host–parasite interplay or host inflammatory response is unclear. Here, we review the neurobiology of helminths, and discuss recent studies on neuropeptide synthesis and function in the helminth as well as the host CNS and immune system. Neuropeptides are summarized according to structure and function, and we discuss the complex enzyme processing for mature neuropeptides, focusing on helminth enzymes as potential targets for novel anthelminthics. We next describe known immunomodulatory effects of mammalian neuropeptides discovered from mouse infection models and draw functional parallels with helminth neuropeptides. Last, we discuss the anti-microbial properties of neuropeptides, and how they may be involved in host–microbiota changes in helminth infection. Overall, a better understanding of the biology of helminth neuropeptides, and whether they affect infection outcomes could provide diagnostic and therapeutic opportunities for helminth infections.

## Introduction

Neurotransmitters are essential for neuronal signal transduction and are released by synaptic vesicles [1]. In contrast with these classically secreted neurotransmitters, there is a group of neuromodulators released ‘extrasynaptically’, which are transported at long distance and exert their effector functions at distant sites. ‘Neuropeptides’ constitute one of the most common, diverse and largest group of neuromodulators [2]. A mature neuropeptide is a short peptide of 3–100 amino acid residues, secreted by different types of cells (mostly of neuronal origin) to perform a variety of functions by binding to a diverse group of G-protein coupled receptors (GPCRs), leading to slow onset but long-lasting synaptic modulation [2,3]. Due to the great diversity in GPCRs and neuropeptides, they can stimulate neurons and have effects on a multitude of tissues (e.g. brain, cardiovascular, respiratory, gastrointestinal) and cells (e.g. immune cells, epithelial cells, neurons) in autocrine, paracrine or endocrine manners [2–4]. Neuropeptides play crucial roles in defining plastic behaviours, learning, memory and immune responses. For instance, neuropeptides are secreted by neuronal cells as signalling molecules that regulate feeding behaviour and sleeping/waking cycles, whereas immune cells can also secrete neuropeptides during infection or inflammation [5,6].

Neuropeptides are categorized into different classes based on their amino acids composition, structure or origin [2,7]. For example, hypothalamic and pituitary neuropeptides are CNS-specific neuropeptides, which include corticotrophin-releasing hormones like somatostatin and alpha-melanocyte stimulating hormones like beta-endorphin, whereas galanin, and neuromedin K are neuron-specific neuropeptides [8,9]. On the other hand, some neuropeptides are secreted by both neuronal as well as immune cells such as neuropeptide Y [10,11].

Due to their diverse functions in different tissues and the advent of -omics tools, neuropeptide research has gained much attention in past few years and their interdisciplinary roles gave birth to a new field known as neuro-endocrinology [12,13]. In vertebrates, several neuropeptides have been studied in detail, and a list of prominent neuropeptides and their tissue specificity is provided in Table 1. Owing to technical constraints and scientific challenges, such as the lack of accessibility of samples, our knowledge of invertebrate's neuropeptides, especially invertebrate helminth parasites, is limited. Among invertebrates, most of our understanding of neuropeptides has involved the nematode *Caenorhabditis elegans* and fly *Drosophila melanogaster* [2,34–36]. Here, we describe the helminth nervous system, summarize what is known of invertebrate neuropeptides based on these studies, and finally extend our discussion to the structure and function of neuropeptides in parasitic helminths, focusing on recent -omics studies.

**Table 1.** Main neuropeptides of vertebrates and orthologs in helminths

Neuropeptide family	Vertebrate tissue expression	Presence in helminths	Function	Receptors in helminths	References
Corticotropin-releasing hormones	Hypothalamus	*C. elegans*	Regulate locomotor activities	*C. elegans*	[15]
Somatostatin	Hypothalamus, pancreas	Unknown	Regulate immune system	*C. elegans*	[15–17]
Neuropeptide Y	Brain, intestine, immune	*C. elegans*	Parasite feeding Host immune regulatory	*C. elegans*	[18–20]
QRFP peptides	Hypothalamus	*Echinococcus* *Ascaris*	Parasite motility	Unknown	[21,22]
Oxytocin/vasopressin	Pituitary	*C. elegans*	Reproductive behaviour Associative learning	*C. elegans*	[23,24]
Opioid peptides	Brain	*S. mansoni* *C. elegans*	Regulate immune system Regulate feeding behaviour	*S. mansoni*	[24–26,28]
Glucagon/secretin	Brain and pancreas	Unknown	Metabolism Immune respinse	Unknown	[27]
Galanin family	Brain	*C. elegans*	Regulate foraging behaviour	*C. elegans*	[29]
Substance P-like tachykinins	Brain	*C. elegans*	Promotion of aggression, sexual activity and fecundity	*C. elegans*	[30]
Serotonin	Brain/gut	*C. elegans*	Feeding, reproduction	*F. hepatica*	[114]
Neuropeptide F	Brain/heart	*S. mansoni*, *F. hepatica*	Myoexcitation	Unknown	[31]
Insulin-like peptides	Brain	*T. solium*, *Echinococcus multilocularis*	Glucose and glycogen metabolism	*T. solium*	[32,115]

### Helminth nervous system

Helminths are considered to have a very simple nervous system which is comprised mainly of neuronal cells and different types of nerve cords to transmit the signal molecules. All helminths share a similar nervous system structure, but very little is known about helminth neurobiology and the soluble factors that govern helminth neuronal function. Helminths are broadly categorized into the phyla Nematodes and Platyhelminthes, where Platyhelminthes are further classified into Cestodes (tapeworms) and Trematodes (flukes). The cestodes and trematodes comprise various infectious parasitic families such as Taeniidae, Hymenolepididae, Dipylidiidae, Diphyllobothriidae and flukes (blood, liver, lung and intestinal), respectively [37,38]. These worms grow in different organs in the host depending on the requirements for their life cycle. For instance, adult tapeworms reside in the intestine while the larval stage parasite grows in other organs [36,39]. Externally, adult tapeworms are differentiated into three body parts: scolex, neck and proglottids (immature and mature). The scolex comprises the nervous system of tapeworms as different nerve cords originate from it and are connected to the cerebral ganglion, which is considered the brain of worm [40–42]. Immunohistochemical studies supported the presence of neuropeptides, such as FMRF-amide, in these nerve cords in adult tapeworm *Echinococcus* [43]. The nervous systems of nematodes have been studied by various research groups, providing insight into its composition. The CNS of nematodes is called the neuropil which comprises nerve ring, cords (dorsal and ventral) and sensory neurons. Neuronal cell bodies are positioned anterior and posterior of the nerve ring; these neuronal cell bodies are also known as ganglia [44].

**Figure 1.**
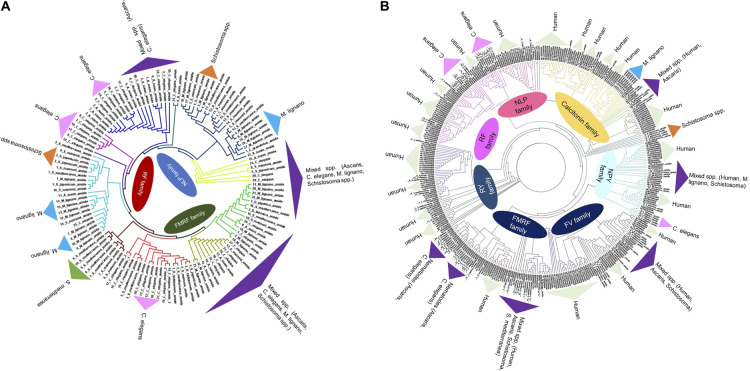
Phylogenetic tree of neuropeptides from different helminthic species such as *C. elegans*, *Schistosoma sp*, *Ascaris sp*, *Schmidtea mediterranea*, *Macrostomum lignano* (1A) and with *Homo sapiens* neuropeptides (1B). The Neighbor-joining trees was constructed using MEGA 5 software with 1000-fold bootstrap re-sampling. The numbers at the nodes of the branches represent the level of bootstrap support for each branch.

Apart from the nerve cords, the nervous system also comprises different types of nerve cells such as neurons, glial cells and muscle tissue in which neurons have neurosecretory function by releasing different neuroactive substances [45]. The larval and adult stage platyhelminthes secrete different types of neuroactive substances such as peptidergic FMRFamide, RYamide and FVamide. These peptides share sequence homology with vertebrate neuropeptides and are cholinergic (serotonin) in nature, where they play major roles in neurotransmission as well as in feeding, and reproduction of the worm. The same neuropeptides expressed by vertebrates were reported to be involved in altering lymphocyte (T-cell) proliferation [46–48]. Phylogenetic analysis between human and helminth's (cestode, trematode and nematode) mature neuropeptides indicate sequence homology (Figure 1). Immunohistochemical studies revealed the presence of different neuropeptides such as FMRF-amide neuropeptide in sensory neurons in *Ascaris* species, whereas FLP neuropeptides were detected in the different organs of the *C. elegans* nervous system, including cephalic papillary nerves, pharyngeal muscles, pharyngeal neurons, etc. [49–51] The nervous system of any parasite is an essential organ for survival, hence an attractive target for an anti-helminthic drug [49,50]. It was observed that inhibition of neuropeptide amidation enzyme by RNA interference (RNAi) prevented the growth of *C. elegans* with lethal and sterile traits [51]. Similarly, inhibition of proprotein convertase enzyme 2 (PC2) in *C. elegans* and *Schmidtea mediterranea* resulted in alteration of reproduction, egg laying, feeding and worm motility [52,53]. Identification of inhibitors for neuropeptide synthesis and processing in parasitic helminths, therefore, provides opportunities for novel anthelminthics.

### Synthesis and structure of mature neuropeptides

**Figure 2.**
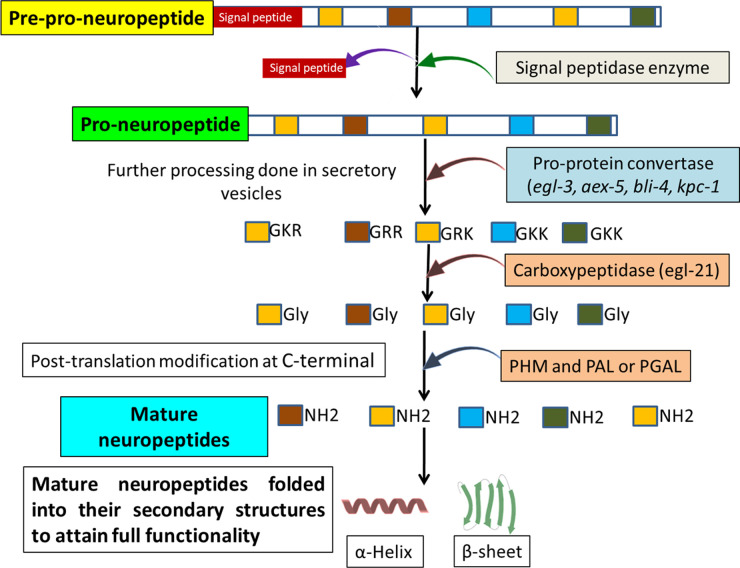
Steps involved in the processing of neuropeptide from pre-pro-neuropeptide to a biologically active mature neuropeptide.

In vertebrates and invertebrates, the protease machinery has a prime role in the processing of large precursor molecules and the formation of these mature neuropeptides. Large precursor proteins are synthesized on ribosomes of the endoplasmic reticulum (ER) in inactive forms which are further processed by proteases and secreted by secretory vesicles of the Golgi apparatus. These large precursor proteins have N-terminal signal peptides, in which the mature neuropeptides are flanking between dibasic residues within the precursor molecule [54,55]. The precursor neuropeptides undergo different modifications in secretory vesicles of the Golgi apparatus and are transported to other organelles based on their fate [56,57]. The multi-step procedure for this complex process is discussed below and depicted in Figure 2.

The N-terminal signal peptide in precursor proteins determines the fate of the proteins in the cell. In helminths, the N-terminal signal peptide comprises positively charged amino acids at their amino terminal, hydrophobic amino acids in the centre and a cleavable part at the carboxyterminal [58]. It provides the signal to the secretory system that these proteins or peptides are transported to their organelles or to the extracellular milieu [59]. The signal peptidase enzymes are serine protease in nature and reside in the membrane of the ER or are packed in secretory vesicles and specifically cleave the signal peptide from newly synthesized proteins [55]. These signal peptidase enzymes are conserved in prokaryotes and in eukaryotes [60].

The next step involves proteolytic cleavage of molecular precursor and subsequent activation. After cleavage of signal peptides, precursor neuropeptides are passed to the secretory vesicles for further processing by different types of proteases (aminopeptidase and carboxypeptidase), known as protein convertases (PC). These enzymes cleave at the dibasic residues present on precursor neuropeptides, which have long, flexible side chains enabling the protease to bind to the substrate. Aminopeptidase and carboxypeptidase cleave at amino (−NH2) terminal and carboxy (−COOH) terminal of dibasic residues, respectively [61]. Along with this, secondary structures around the basic residues also play an important role in specific cleavage by proteases [62,63]. For example, the recognition of the cleavage site of pro-oxytocin is determined by the presence of a β-turn on the N-terminus dibasic (Lys–Arg) residues, this also determines the degree of accessibility and exposure of the processing site [64].

In nematodes such as *C. elegans* and *Ascaris*, different pro-protein convertase enzymes were reported, which mediate cleavage at the dibasic residues of the large neuropeptide precursors [64,65]. In cestodes, there is no direct evidence of proprotein convertase enzymes, but the presence of mature neuropeptides suggests their existence, and future studies identifying these critical enzymes may provide functional targets for parasitic cestodes*.* In *Mesocestoides corti*, the NPF neuropeptide was reported to play a major role in larval motility. NPF neuropeptides have sequence similarity with vertebrate NPY; both are amidated at the C-terminal and the mature neuropeptide flanks dibasic residues in the precursor protein. Immunoreactivity studies showed that NPF neuropeptides are expressed by the whole tapeworm and play important roles in the contraction of nerves in muscle fibres [46,66–68]. In *Monieza expansa*, a sheep tapeworm, the NPF is produced by the adult lifecycle stage and has a myogenic effect on the parasite's muscle contraction. Tapeworms also express other neuropeptides, such as FMRF-amide, vasotocin, neurotensin and leu-enkephalin. The precursor molecules of all these peptides have dibasic residues and are amidated at the C-terminal, which support the presence of pro-protein convertase enzymes in tapeworms [32]. Genomic studies of *Taenia solium* cestode have shown that its larval stage expresses insulin-like peptides (ILP) [69]. The EGL21 enzyme, an analogue of carboxypeptidase E in *C. elegans*, plays an important role in the processing of these neuropeptides [70]. More comparative studies are required to explore the neuropeptide biology in tapeworms and in-silico analysis might provide evidence about the presence of these enzymes.

To gain full functionality, post-translational modifications are required in neuropeptides, including modification at the C-terminal by the two enzymes, peptidyl-α-hydroxylating monooxygenase (PHM) and peptidyl-α-hydroxyglycine-α-amidated lyase (PAL). The carboxyl group of glycine at the C-terminal is ionizable and amidation leads to the non-ionizable form, which enhances the stability and protection of mature neuropeptides against protease activity. In *C. elegans*, mutation in any one of these two enzymes leads to the synthesis of defective neuropeptides [71]. In trematodes such as *Schistosoma mansoni*, amidating enzymes were reported to have significant roles in the growth and survival of the parasite. The RNAi study to knockout the PAL in *S. mansoni* validated the essential role of these enzymes in the survival of the parasite [72]. Either of these enzymes may therefore serve as valuable drug targets, especially since they have no homology with human PHM and PAL enzymes [31,72]. However, there is no direct evidence of their presence in cestodes, and comparative studies to identify these enzymes or functional orthologs may provide a valuable research avenue for new therapeutic targets against these parasites.

Neuropeptides have positive charge (+2 to +6) at neutral pH, which helps them bind to negatively charged cell membranes [73,74]. The FMRFamide and vasoactive intestinal peptide (VIP) also have net positive charge [75,76]. These neuropeptides fold into different secondary structures such as alpha-helix or beta-sheets, based on their amino acids sequence composition [77,78].

### Helminth neuropeptide receptor classification

Neuropeptides bind to GPCRs, but occasionally they also bind to tyrosine receptors for their effector function. Different techniques were employed for the identification of GPCRs in helminths, including mass spectrometry and in-silico screening. In nematodes, two classes of GPCR were reported: first, a rhodopsin class A family receptor (neuropeptide Y/RFamide-like receptors, Somatostatin and galanin-like receptors, Tachykinin (neurokinin)-like receptors, Cholecystokinin/gastrin-like receptors, Gonadotropin-releasing hormone, and oxytocin and vasopressin-like receptors); second, a class B secretin receptor. In Platyhelminthes, studies reported the presence of class A rhodopsin, class B secretin and class E frizzled neuropeptide receptors [79–81].

### Immune modulatory roles of host and helminth neuropeptides

During infection, helminths secrete a variety of biomolecules including proteins, cytokine-like mimics, RNA, lipids and also specific neuropeptides, collectively called helminthic factors. Multiple studies suggest that, instead of mediating effects on themselves, helminths use some of these factors to modulate the host immune system for their own invasion and growth. Helminthic infections also have bystander protective roles against allergic and bacterial infections by inducing higher expression by the host of anti-inflammatory cytokines such as IL-10, transforming growth factor-β (TGF-β) [82–84]. Helminthic factors may exert similar functions to the vertebrate neuropeptides, which exhibit a pleomorphic nature as they play neuronal and immune functions. In neurocysticercosis caused by *T. solium*, substance P-expressing cells were located adjacent to the parasite worm in the brain and caused seizures in wild-type, but not in substance P precursor deficient rats [85]. Apart from its neuronal function, substance P is also expressed by macrophages, monocytes and eosinophils, and increases the production of pro-inflammatory cytokines such as IL-1β and TNF-α along with reactive oxygen species (ROS) production [86,87]. In *T. solium* infection, the inflammatory cytokine response was significantly reduced in mice deficient in substance P and neurokinin [88]. In contrast, somatostatin reduced inflammatory cytokines (IL-1β and IFN-γ) and the granulomatous response to *T. solium* infection. These data reveal distinct pro-inflammatory and anti-inflammatory roles dependent on the neuropeptides. While most data have investigated host-derived neuropeptides, invertebrate orthologs of neuropeptides exist, including substance P, VIP, serotonin, peptide histidine isoleucine (PHI) and peptide YY (PYY), which were reported in the nervous system of *Echinococcus granulosus* metacestode [89,90]. These neuropeptides are encoded and transcribed in a similar manner in both vertebrates and invertebrates, and further studies are needed to investigate whether helminth-derived neuropeptides have similar neuronal and immune functions to their vertebrate counterparts. For instance, VIP secreted by adult flatworms residing in the human intestine may prevent intestinal inflammation. In vertebrates, VIP has immunoregulatory roles, with increase in expression in inflammatory conditions and in autoimmune conditions such as sepsis and rheumatoid arthritis, where it down-regulates the inflammatory response [91,92]. Adult tapeworms such as *Hymenolepis diminuta* exhibited immune cross-reactivity with vertebrate neuropeptides, including pancreatic polypeptide (PP) and FMRFamide neuropeptides, which was observed under immunofluorescence microscopy and radioimmunoassay [93]. In *S. mansoni*, the neuropeptide serotonin was detected in the body wall, where it mediates contraction of body wall muscles and stimulates motor activity. Serotonin had a significant role in the motility of the worm and it acts through the GPCR serotonin receptor (Sm5HTR) [94,95]. Strikingly, the molecular target of praziquantel, the drug to treat schistosomiasis, was shown to be the serotonin receptor where it acted as a partial agonist affecting both the helminth and the host [96]. Given that serotonin receptors have major roles in the movement, development and reproduction of Platyhelminthes, including *E. granulosus* and *M. corti*, they may be broadly relevant drug targets to control platyhelminthic infections. These receptors are also expressed by both larval and adult stages of the parasite, allowing the entire parasite life cycle to be targeted [97,98].

The pro-opiomelanocortin (POMC) derived neuropeptides, including β-endorphin, adrenocorticotropin (ACTH), melanocyte-stimulating hormone (alpha-melanotropin, α-MSH) and met-enkephalin, have been reported to be expressed by all stages of trematode *S. mansoni. S. mansoni*-derived neuropeptides (α-MSH) have been shown to be immunosuppressive. Specifically, parasitic ACTH and α-MSH suppressed lymphocyte responses and the production of IFN-γ and IL-2 [25,99]. Other opioid peptides were also detected in helminths *Diphyllobothrium dendriticum* and *Schistocephalus solidus*. Immunohistochemistry studies showed the reactivity of anti-met-enkephalin, anti-leu-enkephalin and anti-vasotocin sera in different regions of their body. Leu-enkephalin and met-enkephalin are pentapeptides which are processed products of pro-enkephalin A, enkelytin and peptide B. Leu-enkephalin was found in the peripheral nerve net and along the main nerve cords whereas met-enkephalin was found in the main nerve cord and scolex of *D. dendriticum*. Vasotocin was found in the CNS and peripheral nervous system of *D. dendriticum* [100]*.* These two peptides exhibit sequence similarity with their mammalian counterparts, and also show immune cross-reactivity. The potential that these helminth-derived neurotransmitters affect the host nervous system and immune response, including consequences for infection outcomes, therefore, warrants further exploration.

### Nervous system and immune system cross-talk: neuropeptides as intermediaries

Neuropeptides and cytokines act as soluble messengers between the nervous and immune system and play important role in their cross-talk. The neuropeptides released by neurons alter immune cells such as macrophages by binding to specific GPCR-neuropeptide receptors such as neuropeptide Y receptors expressed by macrophages. Innate immune cells in turn initiate the adaptive immune response such as activating T-cells for pathogen-specific effector function and cytokine secretion [101]. The released cytokines can further activate the neuronal cells and induce the physiological symptoms such as epilepsy or seizures [102,103]. Neuropeptides may also directly affect the adaptive immune response in the CNS via T regulatory (T-reg) cells. T-cells also express various neuropeptide receptors for substance P, calcitonin gene-related peptide (CGRP), somatostatin and VIP, and direct interaction with respective neuropeptides of these receptors induces the release of IL-4, IL-10 and IL-2 cytokines [104,105]. In helminth infections of the brain, T-reg cells primarily work to modulate the host immune response and limit inflammation. Here, the released helminthic factors may directly regulate these cells or act through other immune cells, such as dendritic cells and macrophages [105,106]. The NPY is another neuropeptide that has a direct role in the regulation of the immune response and has major roles in the cross-talk between the nervous and immune system. NPY has five receptors (Y1, Y2, Y4, Y5 and Y6). T-cells express only the Y1 receptor, where it has significant effects on the T-cell induced immune response in helminth infections and in the pathogenesis of autoimmune diseases [107,108]. These studies highlight the possibility that helminth infection-induced neuropeptides, secreted by either the host or the parasite, could limit inflammation via T-cells. However, more direct evidence and functional characterization of helminthic neuropeptides, especially NPY, substance P and VIP, which are known to be produced by helminths that infect the CNS, are needed.

### Anti-microbial properties of neuropeptides

Many neuropeptides have anti-microbial activity against a broad range of pathogenic organisms. These include leu-enkephalin and met-enkephalin peptides, which are also expressed by helminths. These peptides have anti-microbial activity against a wide range of pathogens, including viruses, bacteria and fungi [1,109]. Due to their positive charge, they may interact with negatively charged bacterial and other parasitic membrane, which enable them to penetrate through the membrane. Cationic neuropeptides such as VIP and NPY are present in the gastrointestinal tract, oral cavity or beneath the skin, where they have broad antibacterial activity [110]. Anti-microbial peptides (AMPs) were shown to interact with membrane receptors to exert their functions [27]. Helminths, especially those that reside in the intestine, are known to downmodulate the intestinal immune response with protective outcomes against inflammatory bowel disease [111]. There is also increasing evidence that helminths alter the intestinal microbiota by reversing the microbial dysbiosis, and anti-microbial properties of these peptides may be one of the mechanisms of promoting beneficial and diverse microbiota [112,113]. For example, putative anti-microbial neuropeptides have been identified in *Strongyloides* sp., which have been shown to alter the host microbiota in rodent infection models [14]. It is possible that the production of anti-microbial neuropeptides by helminths may contribute to the altered intestinal microbiota through other effector molecules. However, the direct association for alteration in microbial flora and helminth-derived neuropeptides has yet to be demonstrated experimentally.

## Conclusion

**Figure 3.**
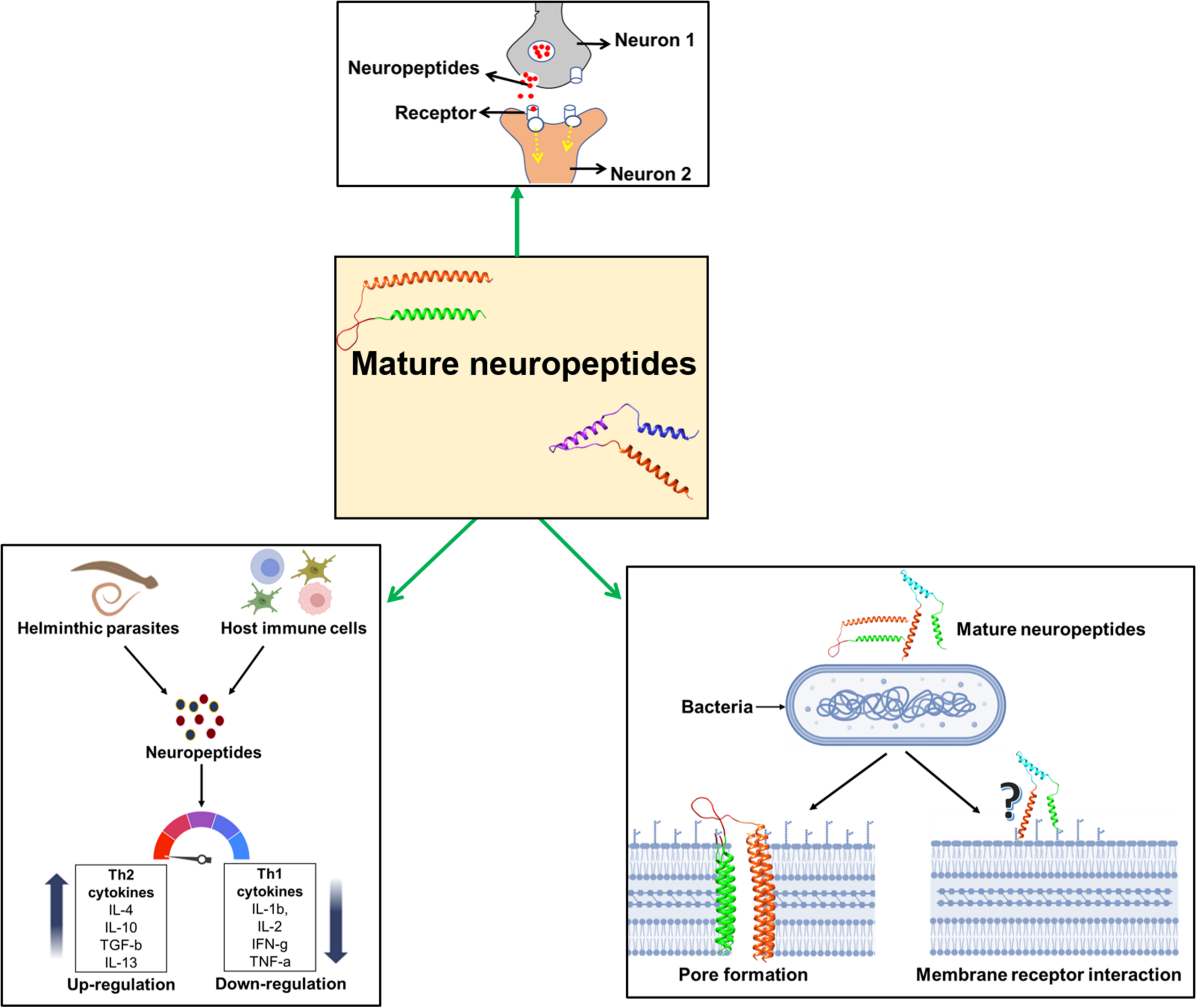
Helminth-derived neuropeptides regulate the neuronal activity, promote Th2 immune responses and exert anti-microbial activity.

Mature neuropeptides are present in different parts of the helminth and play major roles in transferring information between neurons and other cells of the parasite but may also affect the host (Figure 3). Platyhelminthic neuropeptides share sequence similarity with vertebrate neuropeptides, and they competitively bind with their receptors on host cells, perhaps as a mechanism to subvert the host nervous and immune system. Impaired production of neuropeptides by the helminth impairs its growth, yet more research is required to explore potential roles for these helminth-derived peptides in the host and whether they influence host-helminth interaction. Mature neuropeptides are processed by various types of protease enzymes such as signal peptidase, proprotein convertases (aminopeptidase, carboxypeptidase) and post-translational modification enzymes. Many in-silico studies had been undertaken to predict mature neuropeptides and their processing enzymes in different types of invertebrates and predicted their possible roles. More elaborate studies in helminths will be necessary to fully explore the biological roles of neuropeptides and the proteases that process them. A better understanding of these two may provide novel anthelminthic drug targets or allow the discovery of new neuro-immune therapeutic modulators for inflammatory and autoimmune disease.

## Perspectives

- Neuropeptides comprise a diverse and broad group of neurotransmitters in vertebrates and invertebrates, with critical roles in neuronal signal transduction.
- Helminth infections induce neuropeptide production by both host and helminth, with potential consequences for host–parasite interplay and the host inflammatory response.
- Future research into the biology of helminth neuropeptides, and whether they affect infection outcomes, could provide diagnostic and therapeutic opportunities for helminth infections.

## Open Access

Open access for this article was enabled by the participation of the University of California - Riverside in an all-inclusive *Read & Publish* pilot with Portland Press and the Biochemical Society.

## Abbreviations

ACTH: adrenocorticotropin
CNS: central nervous system
ER: endoplasmic reticulum
GPCR: G-protein coupled receptor
GPCR: G-protein coupled receptors
PAL: peptidyl-α-hydroxyglycine-α-amidated lyase
PC2: proprotein convertase enzyme 2
PHM: peptidyl-α-hydroxylating monooxygenase
RNAi: RNA interference
VIP: vasoactive intestinal peptide
VIP: vasoactive intestinal peptide
